# Nanoengineered Chitosan–Genipin Coating of Yeast-Derived Biopolymer Microcapsules for Theranostic Applications

**DOI:** 10.3390/polym18070883

**Published:** 2026-04-03

**Authors:** Beata Miksa, Katarzyna Trzeciak, Slawomir Kaźmierski, Patrycja Przygodzka, Magdalena Ziąbka, Aneta Węgierek-Ciuk, Paulina Blazinska, Damian Mickiewicz

**Affiliations:** 1 Centre of Molecular and Macromolecular Studies, Polish Academy of Sciences, Sienkiewicza 112, 90-363 Lodz, Poland; katarzyna.trzeciak@cbmm.lodz.pl (K.T.); slawomir.kazmierski@cbmm.lodz.pl (S.K.); paulina.blazinska@cbmm.lodz.pl (P.B.); damian.mickiewicz@cbmm.lodz.pl (D.M.); 2 Laboratory of Cellular and Molecular Biology, Institute of Medical Biology, Polish Academy of Sciences, 93-232 Lodz, Poland; p.przygodzka@cbm.lodz.pl; 3 Department of Ceramics and Refractories, Faculty of Materials Science and Ceramics, AGH University, 30-059 Krakow, Poland; ziabka@agh.edu.pl; 4 Institute of Biology, Jan Kochanowski University, 25-406 Kielce, Poland; aneta.wegierek-ciuk@ujk.edu.pl

**Keywords:** chitosan–genipin, bioconjugate, yeast-shell microcapsules, encapsulation of 5-Fluorouracil, polysaccharide carriers for anticancer drugs, theranostic therapy

## Abstract

The development of effective and trackable drug delivery systems remains a major challenge in anticancer therapy. In this study, we designed novel polysaccharide-based theranostic carriers using a yeast-shell (YC) framework, providing a biocompatible platform for intracellular drug delivery. For the first time, a chitosan–genipin bioconjugate was synthesized via a solvent-free, green mechanochemical method and applied as an outer coating to microcarriers encapsulating the anticancer drug 5-fluorouracil (5-FU) and the fluorescent dye phenosafranin. The resulting system enabled simultaneous fluorescence tracking and the controlled release of the chemotherapeutic agent. In vitro evaluation using the MDA-MB-231 triple-negative breast cancer cell line demonstrated that 5-FU retained its antiproliferative activity, while the carriers facilitate sustained intracellular delivery. These findings highlight the potential of YC-based polysaccharide carriers, surface- modified with chitosan–genipin to enhance hydrophilicity, as a versatile platform for anticancer therapy, combining biocompatibility, traceability, and controlled drug release.

## 1. Introduction

**Figure 1:**
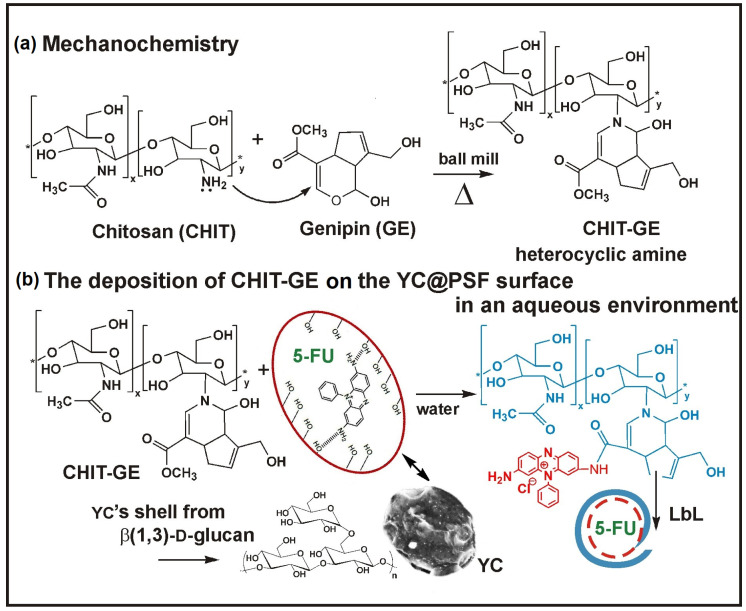
(**a**) The schema of the mechanochemical synthesis of chitosan–genipin (CHIT–GE) bioconjugate. (**b**) Illustration of the deposition of CHIT–GE on YC-shell templates with entrapped phenosafranin (PSF) and 5-fluorouracil (5-FU).

Yeast cell-derived microcapsules (YCs) obtained from *Saccharomyces cerevisiae* [1] are often used as carriers for drug delivery and vaccine development [2,3,4,5,6]. Their hollow and porous microstructures, with a diameter of (2–4) µm and an ellipsoidal shape, are prepared from Backer’s yeast, with an outer cell wall composed primarily of β(1,3)(1,6)-D-glucan and chitin [7,8,9,10]. Different biomolecules and active pharmaceutical ingredients (API) are loaded into the YC-shell via holes with diameters of approximately 500–600 nm [11,12,13]. Moreover, the porous surface of YCs, formed from triple-helical polysaccharide chains within a highly cross-linked glucan network featuring a mesh size of 50–100 nm, enables the adsorption of drug molecules via electrostatic interactions. Additionally, the nonuniform architectural heterogeneity of the YC-shell (diverse spatial organization of β-glucan and chitin) causes its selective phagocytosis. As a result, YC-shell templates are taken up by phagocytic macrophage cells expressing the Dectin-1 receptor, which facilitates the degradation of glucan into smaller fragments subsequently taken up by granulocytes. Soluble β(1,3)-D-glucan binds to the CR3 (CD11b/CD18) receptor on granulocytes, enabling CR3 to mediate leukocyte cytotoxicity against tumor cells coated (opsonized) with iC3b. Endocytosis and transport through the lymphatic system, via an in vivo targeting strategy, enhance the immunogenicity of a cancer vaccine [14,15,16,17]. Therefore, YC-shell containers are of great importance as modern, safe, and selective drug carriers [18,19,20]. However, the burst release of small molecules from YC-shell pores is a disadvantage in long-term anticancer therapy. Such difficulties can be addressed using a layer-by-layer (LbL) synthetic strategy that involves coating the YC-shell with oppositely charged polysaccharides, such as cationic chitosan, followed by anionic alginate, thereby preventing the unpredictable leakage of therapeutics [21,22,23,24,25]. Moreover, natural YC-shell templates demonstrate resistance to elevated temperatures and various environmental stressors, such as organic solvents, while retaining their shape and uniform size. These characteristics of capsular material, including high biocompatibility and biodegradability, make the carriers safe for encapsulating biomolecules and drugs under mild conditions. Chitosan (CHIT) can form the outer layer of the YC-shell, providing a stable matrix for drug delivery applications owing to its amine functional groups. These groups can be chemically and physically modified through ionic and electrostatic interactions, enabling the formation of a fluorescent coating around the capsules [26,27]. In a recent study, a CHIT polymer was substituted with hydrophilic genipin (GE) molecules to obtain a CHIT–GE bioconjugate with photosensitive properties [28,29,30]. Here, we present, for the first time, the synthesis of a bioconjugate in which CHIT is substituted by GE in a non-aqueous environment using a solvent-free, green-chemistry mechanochemical method. The process, driven by mechanical and thermal stimuli and involving heating at 140 °C for 40 min, was carried out in a vibratory ball mill significantly faster than in an aqueous solution with better selectivity [31]. Our current study reports the preparation of the YC-shell co-encapsulating the photosensitive dye phenosafranin (PSF) and 5-fluorouracil (5-FU), which were coated with a bioconjugate CHIT–GE polymer. The naturally occurring YCs containing β(1,3)-D-glucan were enriched with amine groups of PSF, which reacted with CHIT–GE to form a covalently cross-linked network on their surface. A schematic illustration of the synthesis of (YC@PSF)@5-FU(CHIT–GE) microcapsules is shown in Figure 1.

Herein, we report a novel mechanochemical approach for the synthesis of the CHIT–GE bioconjugate via a selective nucleophilic reaction between the amine groups of CHIT and the olefinic carbon atom of GE, leading to the rapid opening of the dihydropyran ring. The resulting CHIT–GE bioconjugate, containing an ester group, acts as an intermediate that, upon hydrolysis, can react with amine-functionalized microcapsule surfaces to form an additional coating layer. This strategy enables improved control over microcapsule structure and surface properties, resulting in enhanced stability.

This work aims to design and characterize polysaccharide-based microcapsules with tunable surface properties for theranostic applications, enabling controlled 5-FU delivery, improved structural stability, and the potential for combined therapeutic and diagnostic use in antiproliferative studies on the MDA-MB-231 triple-negative breast cancer cell line.

## 2. Materials and Methods

### 2.1. Materials

All chemicals used in this investigation were obtained from commercial sources, with the highest available purity. 5-Fluorouracil (5-fluoro-1H,3H-pyrimidine-2,4-dione, 5-FU; purity of 99%), phenosafranin hydrochloride (3,7-diamino-5-phenylphenazinum hydrochloride, PSF; purity of 80%), 4′,6-Diamino-2-phenylindole (DAPI), (3-(4,5-dimethylthiazol-2-yl)-5-(3-carboxymethoxyphenyl)-2-(4-sulfophenyl)-2H-tetrazolium (MTS), chitosan (CHIT, deacetylated chitin, poly(D-glucosamine, with a medium molecular weight 50–1900 kDa, 75–80% degree of deacetylation, viscosity 20–300 cP), and dialysis tubing with a membrane (11,000 Da molecular weight cut-off) were purchased from Merck (Darmstadt, Germany). Genipin (GE, methyl(1S,2R,6S)-2-hydroxy-9-(hydroxymethyl)-3-oxabicyclo [4.3.0]nona-4,8-diene-5-carboxylate, with molecular weight 226.2, purity of 98%) was purchased from Pol-Aura (Dywity, Poland). Commercial baker’s yeast (*Saccharomyces cerevisiae*) was purchased from a local grocery store.

### 2.2. Synthesis of a CHIT-GE Bioconjugate

Milling was performed in 1.5 mL stainless steel jars using three 4 mm diameter balls. The grinding conditions were 90 min at 25 Hz with an external heating temperature of 140 °C. The CHIT/GE mixture (17.50 mg/15.06 mg, *w*/*w*) was prepared with 10 µL of H_2_O as a liquid-assisted grinding (LAG) agent at pH 2.

### 2.3. Procedure for Yeast Cells (YC) Extraction

Generally, the alkaline extraction of yeast cells (40 g) was performed in 400 mL of 10% (*w/v*) sodium hydroxide solution by heating the dispersion at 80 °C under constant stirring for 1 h. The suspension was then centrifuged at 2000× *g* for 10 min, and the resulting pellet was washed twice with deionized water. In the next step, the acidic extraction of the pellet suspension was carried out at pH 4.2 and 55 °C for 1 h. The pellet was further purified using isopropanol (4 × 100 mL) and acetone (2 × 100 mL) and finally dried under vacuum.

### 2.4. Encapsulation of PSF in YCs

Briefly, 0.5 g of lyophilized YCs was suspended in an aqueous solution of PSF (30 mg of dye in 15 mL of distilled water) and incubated for 12 h at room temperature. The YC@PSF dispersion was then centrifuged at 2000× *g* for 10 min, and the resulting pellet was washed until the supernatant became colorless, indicating the complete removal of residual free dye. For quantitative analysis, the encapsulated dye was released from YC@PSF by sonication in methanol for 30 min, followed by centrifugation.

According to the molar extinction coefficient (ε) of PSF in methanol estimated at 529 nm (ε_529 nm_ = 39,900 M^−1^ cm^−1^), the loading of dye was 1.26 mM per gram of YCs. Finally, YC@PSF was dried under vacuum.

### 2.5. Encapsulation of 5-FU in YC@PSF

Briefly, 200 mg of lyophilized YC@PSF was suspended in an aqueous solution of 5-FU (50 mg in 15 mL of distilled water) preheated to 40 °C, and the suspension was then incubated at room temperature for 12 h. The loaded capsules (YC@PSF)@5-FU were separated by centrifugation at 2000× *g* for 10 min to remove the unentrapped drug. Finally, (YC@PSF)@5-FU was dried under vacuum.

The amount of loaded 5-FU was determined spectrophotometrically by measuring the absorbance of the initial drug solution and the first supernatant after encapsulation at 266.5 nm. According to the molar extinction coefficient of 5-FU in distilled water, estimated at 266.5 nm and 25 °C (ε_266.5 nm_ = 6439 M^−1^ cm^−1^), the loading of drug was 2.1 mM per gram of YCs. The encapsulation efficiency of 5-FU in YC@PSF microcapsules was 75%.

### 2.6. CHIT-GE Deposition on (YC@PSF)@5-FU

The CHIT–GE bioconjugate (15 mg) was added to the aqueous suspension of (YC@PSF)@5-FU (50 mg of microcapsules in 10 mL of water). The suspension was incubated at pH 5.0 and 40 °C for 4 h with constant stirring. Then, (YC@PSF)@5-FU(CHI–GE) microcapsules were separated using centrifugation at 2000× *g* for 10 min. The resulting pellet was washed three times with water, then lyophilized. The loading of the drug was 130 mg of 5-FU per 1 g of microcapsules.

### 2.7. Fourier Transform-Infrared (FT-IR) Measurements

FTIR spectra were obtained using a Thermo Scientific Nicolet 6700 FT-IR spectrometer (Thermo Fisher Scientific, Waltham, MA, USA) equipped with a Golden Gate ATR accessory and a DTGS detector. Measurements were performed at a resolution of 2 cm^−1^ and a scan rate of four scans per second. All spectra were manually baseline-corrected.

### 2.8. Solid-State Nuclear Magnetic Resonance (SSNMR) Spectroscopic Characteristics

The structures of YC, YC@PSF, (YC@PSF)@5-FU, and (YC@PSF)@5-FU(CHIT–GE) microcapsules were analyzed by solid-state ^13^C cross-polarization magic-angle spinning nuclear magnetic resonance (SS ^13^C CP/MAS NMR) spectroscopy. Experiments were conducted on a Bruker Avance III 400 spectrometer (Bruker, Billerica, MA, USA), operating at 400.13 MHz for ^1^H and 100.63 MHz for ^13^C. A MAS probe with a 4 mm ZrO_2_ rotor was employed, and all spectra were acquired at room temperature with a spinning rate of 8 kHz.

### 2.9. UV-Vis Absorption and Fluorescence Studies

UV-vis absorption spectra were recorded using a Specord S600 diode-array spectrophotometer (Analytical Jena, Jena, Germany) with a 1 cm optical pathlength cuvette. The fluorescence spectra were obtained using an HORIBA (Jobin Yvon) fluorimeter (HORIBA, Kyoto, Japan). All fluorescence analyses were performed in standard 10 mm path length quartz cuvettes. The excitation and emission slits were set to 5.0 nm.

### 2.10. Determination of the Hydrodynamic Diameter and Zeta Potential of Microcapsules

The hydrodynamic diameters of YCs, YC@PSF, (YC@PSF)@5-FU, and (YC@PSF)@5-FU(CHIT–GE) were determined by dynamic light scattering (DLS) using an angle of 173°. Mark–Houwink parameters were used to calculate molecular weight via the general DLS option. Measurements were performed with the microparticles dispersed in a 1.0 mM aqueous NaCl solution at 25 °C. Data were analyzed using a cumulative analysis method, with three independent measurements, each consisting of 11 runs. All experiments were performed in triplicate.

Zeta potentials of the microcapsules in aqueous dispersion at 25 °C were determined from electrophoretic mobility measurements according to the Smoluchowski equation. Measurements were performed with a DTA1060C cell on a Zetasizer Nano Z instrument (Malvern Instruments, Malvern, UK). Data were obtained from three independent analyses, each consisting of 12 runs, and are presented as the mean ± standard deviation (SD).

### 2.11. Nitrogen Adsorption–Desorption Studies

Nitrogen adsorption measurements were performed at −196 °C using a Micrometrics ASAP 2020 Plus analyzer (Micrometrics, Norcross, GA, USA). Samples were degassed under vacuum at 25 °C for 24 h using a FloVac Degasser (Micrometrics, Norcross, GA, USA) before analysis. The specific surface area was calculated according to the Brunauer–Emmett–Teller (BET) theory. Multi-point BET surface areas were obtained from the adsorption isotherms in the relative pressure (P/P_0_) range of 0.10–0.30.

### 2.12. Thermogravimetric Analyses

Thermogravimetric analysis (TGA) of YC@PSF, and (YC@PSF)@5-FU(CHIT–GE) were performed using a TGA 5500 analyzer (TA Instruments, New Castle, DE, USA) under a nitrogen atmosphere. Samples were heated from 0 to 800 °C at a rate of 10 K min^−1^.

### 2.13. The Contact Angle Analysis

The aqueous suspension of microcapsules at a concentration of 25 mg/mL was deposited onto a Petri dish and dried under mild conditions at 30–40 °C. Upon drying, the suspension formed a thin film, which was carefully peeled from the support. Surface wettability, interfacial tension, and surface energy were determined using an SEO-Phoenix Automatic Contact Angle Goniometer (Surface Electro Optics, Suwon, Republic of Korea) operated with SurfaceWare software (version 7.0).

### 2.14. Characterization of (YC@PSF)@5-FU(CHIT–GE) by Confocal Laser Scanning Microscopy (CLSM)

The morphology and internal structure of the (YC@PSF)@5-FU(CHIT–GE) microcapsules were investigated using the confocal microscope (Nicon D-Eclipse C1) and analyzed with EZ-C1 version 3.6 software (Nikon, Tokyo, Japan). For image acquisition, the microcapsules in the storage solution (deionized water) were directly placed in a chambered coverglass system. Two separate fluorescence channels, namely, green (FTIC, excitation at 488 nm) and red (RBITC, excitation at 514 nm), were used to characterize CHT–GE and PSF deposition, respectively.

### 2.15. In Vitro Drug Release

Drug release studies were carried out using (YC@PSF)@5-FU(CHIT–GE) microcapsules dispersed in distilled water at pH 2.0 (adjusted in HCl), 5.5, and 9.0 (adjusted with NaOH). A portion of (YC@PSF)@5-FU(CHIT–GE) (12 mg, corresponding to a loading of 100 mg of 5-FU per gram of microcapsules) was suspended in 4 mL of deionized water adjusted to the respective pH, and placed into a dialysis bag with a molecular weight cut-off of 11 kDa. The sealed dialysis bag was immersed in 50 mL of a release medium contained in glass vials. The vials were maintained at 37 °C in a shaking bath with continuous agitation at 100 rpm. At predetermined time intervals, 1 mL of the external medium (dialysate) was withdrawn to determine the amount of released 5-FU. After analysis, the sample volume was returned to the vial to preserve constant release conditions. The concentration of 5-FU was quantified by UV-Vis spectrophotometry at 266.5 nm. Drug release at different pH values was evaluated and expressed as the percentage of 5-FU released (DR), calculated using the equation below:

DR% = [(5-FU release at *T*_min_.)/5-FU release at *T*_min_. = 0)] × 100%

### 2.16. Microscopic Observation of the Cellular Uptake of Microcapsules, and Cytotoxicity Assays

The morphological changes and intracellular localization of microparticles were assessed in the MDA-MB-231 triple-negative breast cancer cell line. Cells cultured on round coverslips were treated with microcapsules at a concentration of 200 μg/mL, then incubated at 37 °C for 72 h. Following incubation, cells were washed three times with PBS to remove residual culture medium and uninternalized microcapsules. The cells were then fixed in 4% paraformaldehyde for 30 min and washed three times with PBS. Subsequently, a DAPI solution (5 µg/mL) was applied, and the samples were incubated at room temperature for 15 min to stain the nuclei. DAPI binds preferentially to A-T-rich regions of DNA, enabling the visualization of cell nuclei. Finally, the samples were mounted on microscope slides with coverslips, and fluorescence images were acquired for analysis. Microscopic observation was performed using a Nikon Eclipse 80i fluorescence microscope under 20× magnification (Nikon NIS Elements D 3.10 software (Nikon Instruments Inc., Melville, NY, USA)). The filter sets used were TEXAS RED (Ex 560/20, DM 585, Em 605/60) and DAPI (EX 365/28, DM 405, Em 445/50).

The cytotoxic effects of the microcapsules and the crude 5-FU were assessed in vitro using the MDA-MB-231 breast cancer cell line with a quantitative colorimetric assay based on tetrazolium salt reduction. This assay measures the conversion of MTS (3-(4,5-dimethylthiazol-2-yl)-5-(3-carboxymethoxyphenyl)-2-(4-sulfophenyl)-2H-tetrazolium) into a purple formazan product in the presence of phenazine methosulfate. Cells were harvested and resuspended in MEM medium supplemented with 10% (*w*/*v*) fetal bovine serum (FBS) and 1% (*w*/*v*) antibiotics/antimycotics. They were seeded in 96 well-plates at a density of 5.0 × 10^4^ cells per 200 µL per well, and incubated at 37 °C in a humidified atmosphere containing 5% CO_2_ for 24 h. Crude 5-FU was dissolved in distilled water to prepare solutions at the desired concentrations, and microcapsules were similarly suspended in distilled water at corresponding concentrations. After the initial incubation, the medium was replaced with fresh medium containing the test solutions, and cells were further incubated for 72 h. Untreated cells were used as a positive control (100% availability), while medium without cells served as a background control. Following treatment, 20 µL of MTS reagent was added to each well, and the plates were incubated for 4 h at 37 °C. Absorbance was measured at 490 nm using a TECAN Spark Microplate Reader (TECAN, Männedorf, Switzerland). All treatments were performed in triplicate within the same experiment.

### 2.17. Statistical Analysis

All data were expressed as mean ± standard deviation (SD), n = 3. One-way analysis of variance (ANOVA) was used to determine differences among groups, and unpaired Student’s *t*-test vs. control untreated cells, where *p* < 0.05 values were assumed as statistically significant in all cases. The results were analyzed statistically with Microsoft Office Excel 2010.

## 3. Results and Discussion

### 3.1. Structural Analyses of Microcapsules Using NMR and FTIR Methods

#### 3.1.1. NMR and FTIR Spectra of the Synthesized CHIT–GE Bioconjugate

**Figure 2:**
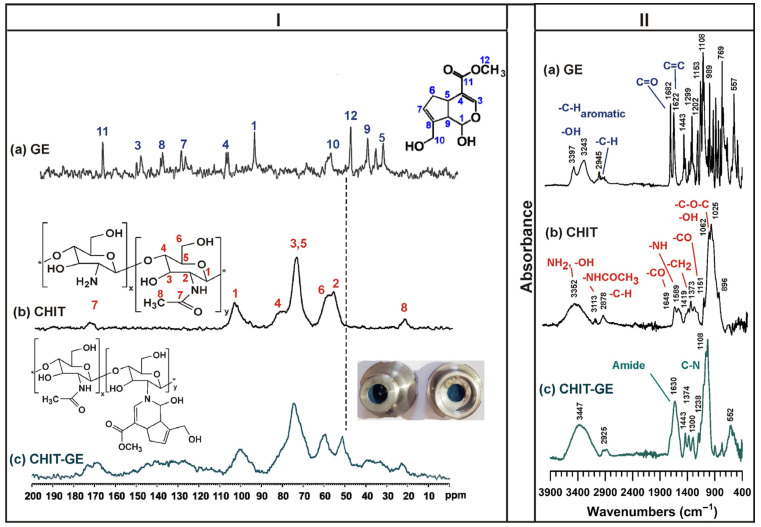
Panel (**I**) illustrates the ^13^C SS NMR MAS spectra of (**a**) genipin (GE), (**b**) chitosan (CHIT), and (**c**) the CHIT–GE conjugate. Panel (**II**) shows the FTIR spectra of (**a**) GE, (**b**) CHIT, and (**c**) the CHIT–GE conjugate.

The chemical structure of the resulting CHIT–GE product was confirmed by ^13^C SS NMR MAS and FTIR spectroscopy (Figure 2I,II, respectively). GE reacts spontaneously with primary amine groups of biomaterials, and the reaction is identified by the formation of blue products [32,33]. Solid-state ^13^C NMR spectra of CHIT and CHIT-GE were compared in Figure 2I. ^13^C signals of native CHIT: 106.29, 99.40 (C1), 58.68 (C2), 83.57 (C4), 76.56 (C5, C3), 62.18 (C6), 23.80 (C8), and 175.3 (C7) had a significant difference from CHT–GE. The observed resonance at 170–180 ppm, corresponding to the amide bonds, would be assigned to the reaction between amine groups on CHIT and the ester group of GE. In the range of 80–87 ppm, the resonance due to C4 disappears after substituting CHIT with GE. It indicates that the CHIT linear structure has changed its conformation. The residual signal of C6 is observed as a shoulder on the C2 resonance and describes the transfer of C2 carbon atoms of the glucosamine unit to chemical bridges. The resonance of C6 carbons is hidden within the broad resonance of C3, C4, and C5, centered at approximately 70–75 ppm. The slight upfield shift in C1 resonance from 102.5 ppm to a broad resonance centered at 100 ppm is caused by the ring current of the heterocyclic aromatic bound to C2 [34]. FTIR spectra illustrated in Figure 2II confirmed the reaction between CHIT and GE. The amine groups of CHIT initiate nucleophilic attack on the olefinic carbon atom at C3 of GE, leading to the opening of the dihydropyran ring, followed by a series of reactions that yield nitrogen iridoid aromatic intermediates and highly conjugated heterocyclic CHIT–GE derivatives [35].

The characteristic absorption bands of ureacted GE appear at 989 cm^−1^, 1682 cm^−1^, and 1622 cm^−1^, corresponding to the C–H ring out-of-plane band, the stretching vibrations of the methoxycarbonyl –COOCH_3_ group, and the C=C bond in the dihydropyran ring, respectively [36]. Additionally, the C–O–C asymmetric stretch and the CH_3_ bend of the methyl ester were observed at 1299 cm^−1^ and 1443 cm^−1^, respectively. Absorption at 1108 cm^−1^ is assigned to vibrations of the cyclic ether. The native CHIT exhibited characteristic absorption bands at 1649 cm^−1^, corresponding to the C=O stretching vibration of amide-I, and at 1588 cm^−1^, assigned to the N–H bending (deformation) vibration of the primary amine NH_2_ group [37]. Other bands observed for CHIT are at 3352 cm^−1^ related to –OH stretching, at 2878 cm^−1^ assigned to the CH stretching, at 1419 cm^−1^ related to the CH_2_ bond, at 1373–1257 cm^−1^ associated with the CN bond, at 1151 cm^−1^ related to the stretching of the C–O–C bond of glucose and β 1-4 bands at 1062 and at 1025 cm^−1^ corresponding to the vibration of the angular deformation of the amino group. In the FTIR spectrum of the CHIT–GE bioconjugate, the band around 1630 cm^−1^ is attributed to the formation of new amide linkages [38]. The band at 1108 cm^−1^ was assigned to the C–N stretch of the tertiary aromatic amine of the cross-linked genipin nitrogen iridoid covalently bound to CHIT [39]. In addition, the relative increase in C–H stretching at 2925 cm^−1^ and the appearance of the following bands, C–N stretching (amide III) at 1238 cm^−1^, and C–H bending of C=C at 878 cm^−1^, were detected. Moreover, the spectrum of the CHIT–GE bioconjugate shows a drastic reduction in the intensity of C=O stretching from GE at 1682 cm^−1^ and the newly formed bend at 1300 cm^−1^, suggesting the presence of heterocyclic amine ring-stretching, which formed through nucleophilic attack by the amine group of CHIT on the olefinic carbon atom at C3 of GE. Furthermore, the color changed from white (CHIT) to blue (CHIT–GE). These results confirmed the formation of covalent CHIT–GE bonds.

#### 3.1.2. NMR and FTIR Spectra of (YC@PSF)@5-FU(CHIT-GE) Microcapsules

**Figure 3:**
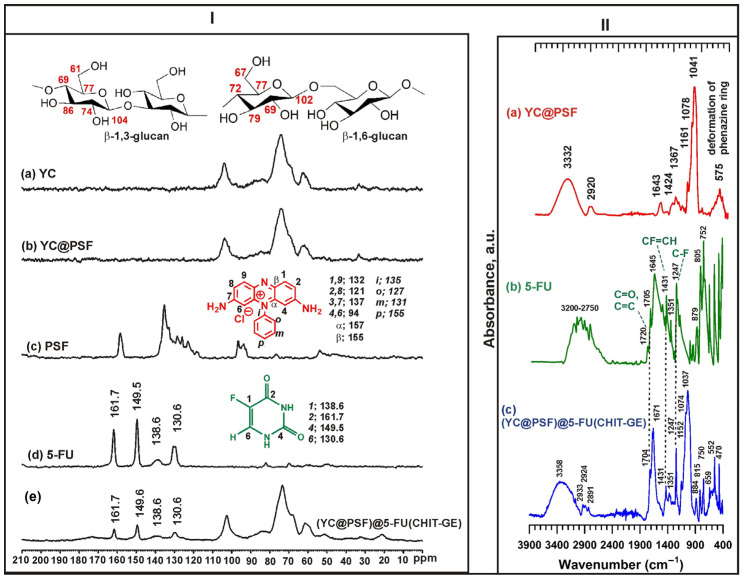
Panel (**I**) illustrates the ^13^C SS NMR MAS spectra of (**a**) YC, (**b**) YC@PSF, (**c**) PSF, (**d**) 5-FU, and (**e**) (YC@PSF)@5-FU(CHIT–GE). Panel (**II**) shows the FTIR absorbance spectra of (**a**) YC@PSF, (**b**) 5-FU, and (**c**) (YC@PSF)@5-FU(CHIT–GE).

The ^13^C SS NMR MAS spectra of the (YC@PSF)@5-FU(CHIT-GE) microcapsules and the corresponding FTIR absorbance spectra are shown in Figure 3, panels (I) and (II), respectively. The CHIT–GE polymer was used to directly coat the YC-shell coencapsulating phenosafranin (PSF) and 5-fluorouracil (5-FU) via the layer-by-layer (LbL) method, a standard procedure for the alternate deposition of polycations and polyanions on yeast-based delivery systems [40]. The successful synthesis of (YC@PSF)@5-FU(CHIT–GE) and its chemical structure was confirmed by ^13^C SS NMR MAS and FTIR spectra, shown in Figure 3I,II. In the FTIR spectrum of (YC@PSF)@5-FU(CHIT–GE), the absorption bands at 1247 cm^−1^ and 1431 cm^−1^ corresponding to C–F and CF=CH stretching vibrations, respectively, indicate the presence of 5-FU. Moreover, carbonyl groups of 5-FU are observed in the ^13^C SS NMR MAS spectrum at 161.7 ppm and 149.6 ppm. The bands at 138.6 ppm and 130.5 ppm are attributed to C–F and C=C in the 5-FU ring, respectively. Additionally, under aqueous conditions, the carboxymethyl groups of GE in the CHIT–GE polymer were hydrolyzed, and the signal at 58.68 ppm almost disappeared in the (YC@PSF)@5-FU(CHI-GE) spectrum. Furthermore, in the FTIR spectrum, the appearance of a new band at 1671 cm^−1^ confirms the formation of a new bond assigned to secondary amide [41]. The substitution of the primary amine group of PSF by GE after the hydrolysis of its ester group led to the covalently bound CHIT-GE layer on the YC@PSF surface.

### 3.2. Morphology of Microcapsules

#### 3.2.1. SEM Images of Pretreated YCs, YC@PSF, and (YC@PSF)@5-FU(CHIT–GE)

**Figure 4:**
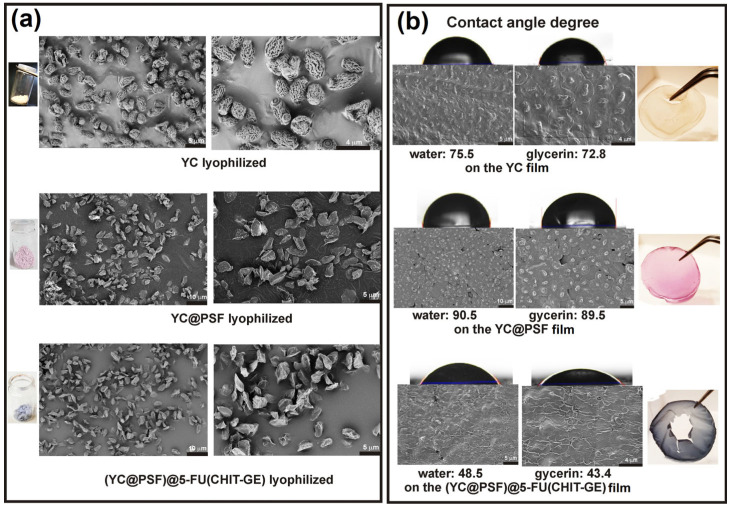
The representative micrographs of lyophilized YCs, YC@PSF, and (YC@PSF)@5-FU(CHIT–GE) microcapsules (**a**), and (**b**) the sample prepared from water suspensions after drying. The images were acquired using scanning electron microscopy (SEM, ThermoFisher Scientific Apreo, Waltham, MA, USA).

The morphology of the pretreated YC–shell, microcapsules with the entrapped phenazine dye YC@PSF, and (YC@PSF)@5-FU(CHIT–GE) with the CHIT–GE coating was examined using scanning electron microscopy (SEM). The micrographs in Figure 4a show the lyophilized microcapsules. Images in Figure 4b were recorded from a water suspension of microcapsules after air-drying. The diameter of (YC@PSF)@5-FU(CHIT–GE) microcapsules was higher than that of the YC-shell and YC@PSF, which exhibited approximately 3.5–4.0 μm (±100 nm), 3.0–3.5 μm (±100 nm), and 4.5–5 μm (±100 nm), respectively. The sample of the lyophilized YC-shell presented a lamellar structure of β(1,3)-D-glucan, indicating a different morphology from microcapsules coated with a CHIT–GE layer. The surface of YC@PSF is smoother than that of the YC-shell, and molecules of the entrapped PSF dye are visible as dots. Moreover, the diameter of YC@PSF decreased because the primary amine groups of the cationic phenazine dye can interact with the hydroxyl groups of YCs, and electrostatic forces cause microcapsules to shrink. Thus, PSF molecules contributed to the stabilization of YCs by ionically cross-linking and forming hydrogen bonds on their surface. As shown in Figure 4b, the surface morphology of (YC@PSF)@5-FU(CHIT–GE) microcapsules confirms the formation of an outer wall by the CHIT–GE layer via GE-mediated extensive cross-linking networks. Furthermore, the structural integrity was maintained, with no visible pores and no evidence of a triple-helix conformation on the surface.

As seen in Figure 4b, the surface of (YC@PSF)@5-FU(CHIT–GE) became rougher, and more creases and folds were found. Their diameter, approximately 5.0–5.5 μm, was also larger than that of YC@PSF. Additionally, images of (YC@PSF)@5-FU(CHIT–GE) reveal a distinct surface microarchitecture composed of domains attributed to the CHIT–GE network, with fibrous, chain-like structures surrounding the microcapsules and distributed across their surface. Furthermore, the SEM images shown in Figure 4b demonstrated that the (YC@PSF)@5-FU(CHIT–GE) structures are slightly convex.

#### 3.2.2. CLSM Images of (YC@PSF)@5-FU(CHIT–GE)

**Figure 5:**
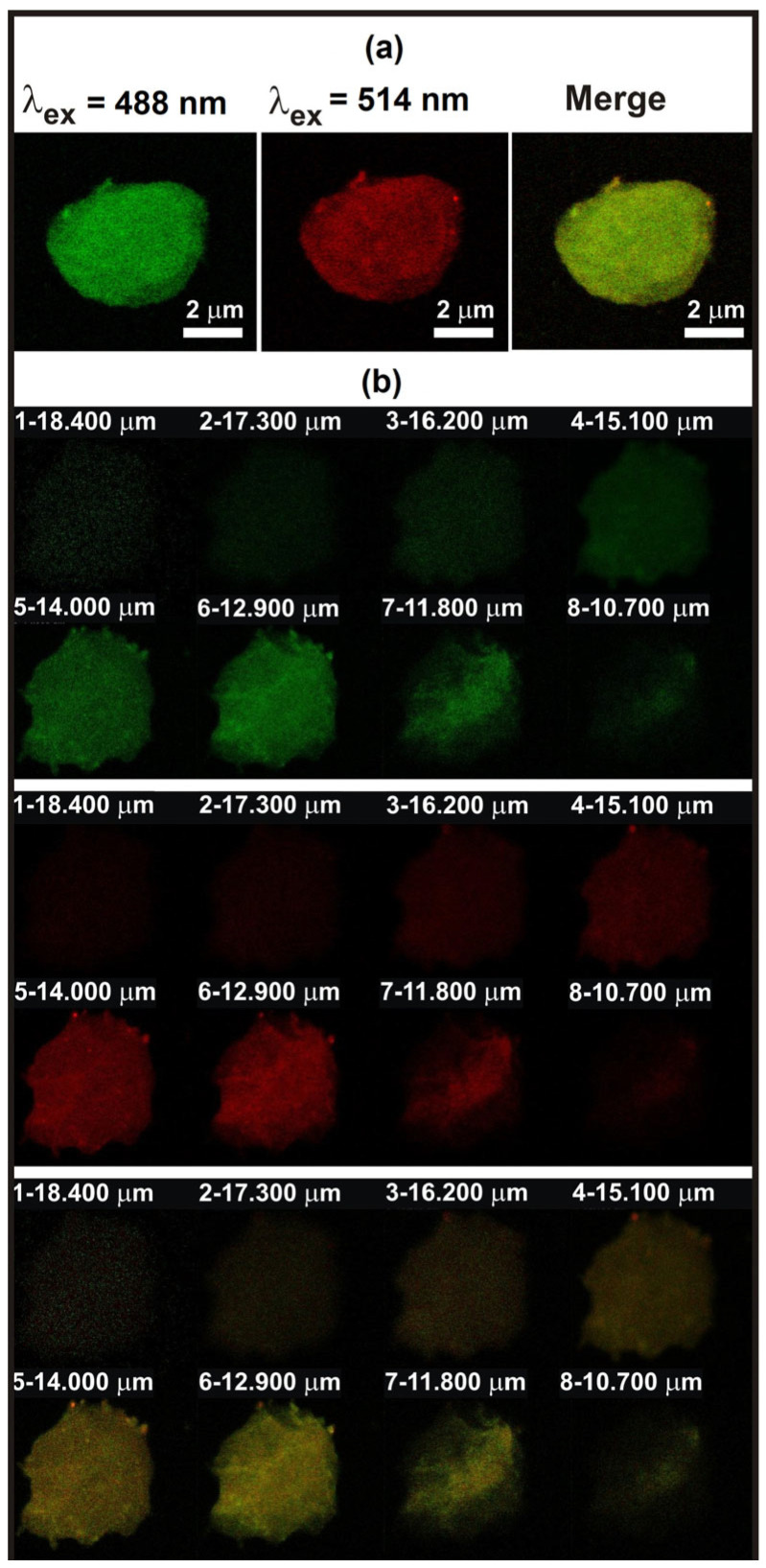
(**a**) CLSM images of (YC@PSF)@5-FU(CHIT–GE) obtained in the green fluorescence mode (λ_ex_ = 488 nm), the red fluorescence mode (λ_ex_ = 514 nm), and as a merged image. (**b**) Illustrates fluorescence distribution across the microcapsule’s wall. Images were acquired at 60× magnification; the scale bar is given in µm.

The fluorescence intensity observed through the microcapsule wall is shown in Figure 5. CLMS was used to obtain sequential optical slices of fluorescently labeled microcapsules, enabling the visualization of the fluorescence distribution across the (YC@PSF)@5-FU(CHIT–GE) wall. Two separate fluorescence channels, namely, green (FTIC, excitation at 488 nm) and red (RBITC, excitation at 514 nm), were used to characterize CHT–GE and PSF deposition, respectively. Eight cross-sections through the capsule wall were obtained to generate a responsive image, as illustrated in Figure 5b. The presence of CHIT–GE coating was confirmed by fluorescence spectroscopy. The CHIT–GE layer was deposited on the (YC@PSF)@5-FU surface via electrostatic interactions, hydrogen bonding, and covalent amide bonds formed between PSF molecules and GE moieties. Neither the PSF nor the CHIT–GE fluorescent components exhibited changes in their emission spectra in (YC@PSF)@5-FU(CHIT–GE). Thus, the red fluorescence of PSF incorporated within the YC wall was not quenched by the CHIT–GE coating. The CHIT–GE coating on the microcapsule surface was clearly identified by the appearance of green fluorescence resulting from the reaction between CHIT and GE. A homogeneous distribution for both photosensitive PSF dye molecules and the CHIT–GE layer was observed across the microcapsule wall. Therefore, the CHIT–GE layer was homogeneously deposited on the microcapsule surface, forming a cross-linked, shell-like membrane. Variations in fluorescence signal intensity, ranging from weak to strong, depend on the thickness of the probed layer and confirm the hollow structure of the microcapsules. Thus, CLSM images of (YC@PSF)@5-FU(CHIT–GE) microcapsules confirmed their structural organization.

### 3.3. Physical Characteristics of (YC@PSF)@5-FU(CHIT–GE)

#### 3.3.1. Microcapsule Size Distribution and Zeta Potential Analysis

**Figure 6:**
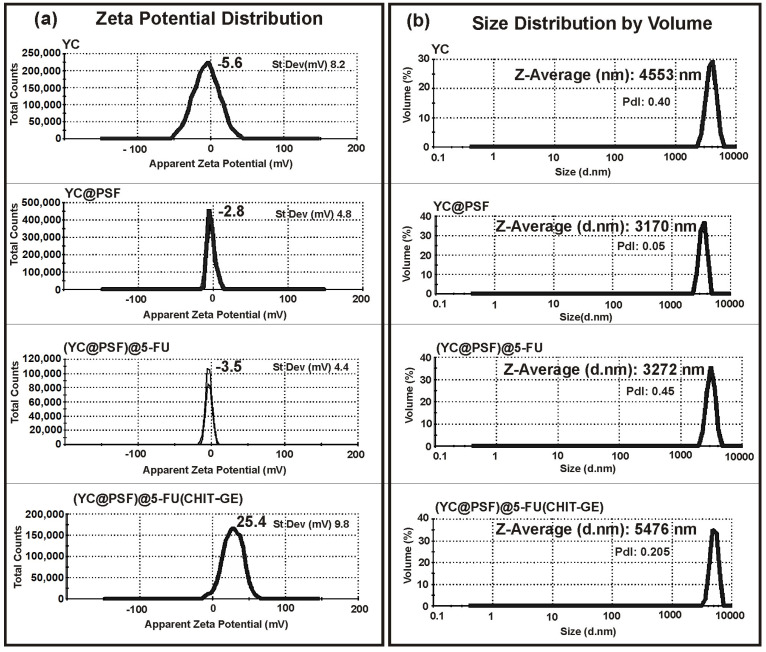
(**a**) Zeta-potential measurements of YCs, YC@PSF, (YC@PSF)@5-FU, and (YC@PSF)@5-FU(CHIT–GE) microcapsules; (**b**) size distribution determined by DLS.

DLS measurements presented in Figure 6a show monomodal size distribution profiles for the YC–shell, YC@PSF, and (YC@PSF)@5-FU microcapsules, with dominant peaks at 4553 nm, 3170 nm, and 3272 nm, respectively. In contrast, (YC@PSF)@5-FU(CHIT–GE) exhibited the largest average diameter (5476 nm), which can be explained by the formation of an additional outer CHIT–GE layer. The assays shown in Figure 6a confirmed changes in the electrical charge of the YC shell after modifications. The zeta potential data indicated that the pretreated YCs initially had a negative charge of approximately −5.9 mV due to their primary composition of β(1,3)-D-glucan.

Entrapping positively charged PSF molecules increased the zeta potential of YC@PSF to −2.8 mV, suggesting electrostatic interactions with the oppositely charged YC wall. Further incorporation of 5-FU into YC@PSF shifted their zeta potential to −3.5 mV. The presence of CHIT–GE coating on the (YC@PSF)@5-FU was evidenced by an increase in zeta potential to 25.4 mV. The stability of microcapsules over time was tested in water dispersion at 37 °C, and the results are displayed in Table 1.

The microcapsules remained stable in aqueous suspension. After 24 h of incubation at 37 °C, YC, YC@PSF, and (YC@PSF)@5-FU showed a slight increase in size due to swelling. Over 72 h, all samples exhibited time-dependent size changes. Native YC decreased from 4891 µm to 4003 µm, while YC@PSF and (YC@PSF)@5-FU showed a smaller reduction, indicating improved structural stability after PSF incorporation and drug loading. In contrast, (YC@PSF)@5-FU(CHIT–GE) displayed the largest sizes (5408–5145 µm) with minimal variation, confirming the formation of a stabilizing CHIT–GE coating. The zeta potential analysis revealed that (YC@PSF)@5-FU showed a more negative value at 24 h (−10.4 mV), which gradually decreased to approximately −4.3 mV, likely due to partial drug release. In contrast, (YC@PSF)@5-FU(CHIT–GE) showed near-neutral values (+0.58 to −1.08), indicating effective surface modification by chitosan. This shift toward neutrality is attributed to protonated amine groups and possible Schiff base formation with released 5-FU. Overall, the results confirm successful LbL assembly, leading to enhanced stability and tunable surface properties.

#### 3.3.2. Contact Angle Measurements

We investigated the surface wettability of YC, YC@PSF, and (YC@PSF)@5-FU(CHIT–GE) microcapsules. The studies measured the contact angles (θ) of water and glycerol droplets on films formed from aqueous suspensions of the microcapsules. The results showed that the film derived from YC@PSF exhibited the highest contact angles, measuring 90.5 ± 2° for water and 89.5 ± 2° for glycerol. This indicates that the YC@PSF surface was the most hydrophobic, likely due to electrostatic interactions as well as hydrogen bonding or possible covalent linkages between the primary amine groups of PSF and the hydroxyl groups of β(1,3)-D-glucan. In contrast, the film derived from (YC@PSF)@5-FU(CHIT–GE) exhibited the lowest contact angles, measuring 48.5 ± 2° for water and 43.4 ± 3° for glycerol, indicating higher hydrophilicity. These findings are consistent with values reported in the literature for chitosan cross-linked with genipin [42]. The experimental results are presented in Figure 4b and summarized in Table 2.

Overall, the physicochemical characterization confirms that each modification step significantly influences the properties of the microcapsules. YC@PSF exhibited the highest hydrophobicity, as evidenced by the largest contact angles and relatively stable microcapsule size, indicating enhanced structural integrity after PSF incorporation. In contrast, (YC@PSF)@5-FU(CHIT–GE) showed increased microcapsules size, near-neutral zeta potential, and markedly reduced contact angles, confirming successful surface functionalization with a hydrophilic CHIT–GE coating. These results demonstrate that LbL assembly enables the precise tuning of surface charge, wettability, and structural stability, which are critical parameters governing drug release behavior and interactions with biological systems.

#### 3.3.3. TGA and Nitrogen Adsorption–Desorption Measurements

**Figure 7:**
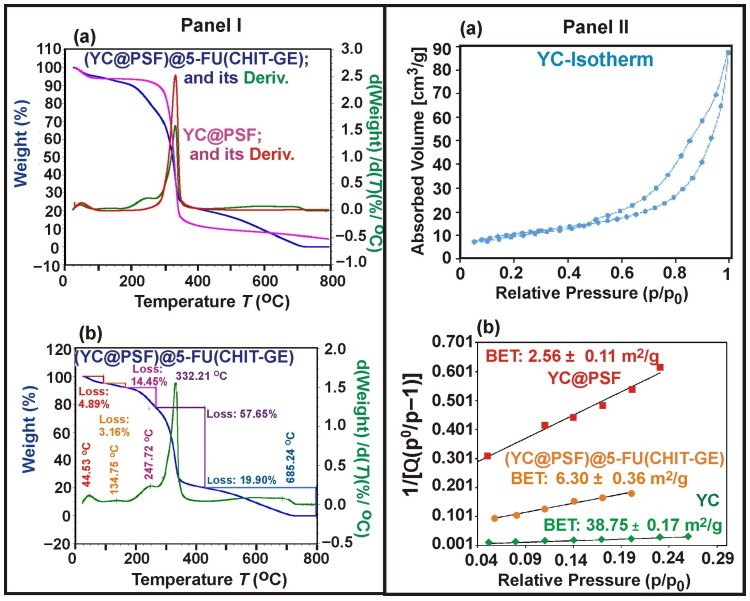
(**I**) (**a**) TGA thermograms of YC@PSF and (YC@PSF)@5-FU(CHIT–GE). (**b**) The derivative thermogravimetric of (YC@PSF)@5-FU(CHIT–GE). (**II**) (**a**) Nitrogen adsorption–desorption isotherm of YC. (**b**) BET surface area measurements for YC, YC@PSF, and (YC@PSF)@5-FU(CHIT–GE) were 38.75 m^2^g^−1^, 2.56 m^2^g^−1^, and 6.3 m^2^g^−1^, respectively.

TGA measurements presented in Figure 7(Ia), revealed distinct differences in the microcapsules’ thermal behavior, confirming the successful synthesis of (YC@PSF)@5-FU(CHIT–GE). The first stage (20–100 °C) corresponds to a 4.8% mass loss, attributed to the desorption and removal of physically adsorbed water. The major mass loss of (YC@PSF)@5-FU(CHIT–GE), approximately 75.3% in the temperature range of 100–425 °C, is associated with the thermal degradation of functional groups (–OH, –COOH), dehydration of the saccharide rings, and cleavage of C–O–C glycoside bonds in the polymeric components [43]. In a nitrogen atmosphere, non-oxidative thermal degradation occurs, including chitosan deacetylation, resulting in a broader degradation region of up to 425 °C. The degradation of chitosan is initiated by the disruption of hydrogen bonds between the N-acetyl and free amine groups. Notably, the decomposition of hybrid (YC@PSF)@5-FU(CHIT–GE) microcapsules begins at slightly higher temperatures than that of YC@PSF. The final stage (425–800 °C), with a mass loss of 19.9%, is attributed to the further degradation of the intermolecular cross-linked CHIT–GE layer deposited on YC@PSF microcapsules, as well as the decomposition of carbonaceous residue and volatile products, including acetic, butyric acids, and other low-molecular-weight fatty acids [44]. Nitrogen adsorption–desorption N_2_ isotherms for the parent YCs, YC@PSF, and (YC@PSF)@5-FU(CHIT–GE) showed a type H3 hysteresis, and results of the calculated specific surface area using the standard BET method are illustrated in Figure 7(IIb). The surface area for parent YCs was 38.75 m^2^g^−1^ and decreased to 6.3 m^2^g^−1^ for (YC@PSF)@5-FU(CHIT–GE).

The reduction in BET surface area, accompanied by a proportional decrease in pore volume, indicates the successful deposition of CHIT–GE on the microcapsule surface.

### 3.4. In Vitro Studies

#### 3.4.1. UV-Vis Spectrophotometric and Fluorescence Analyses

**Figure 8:**
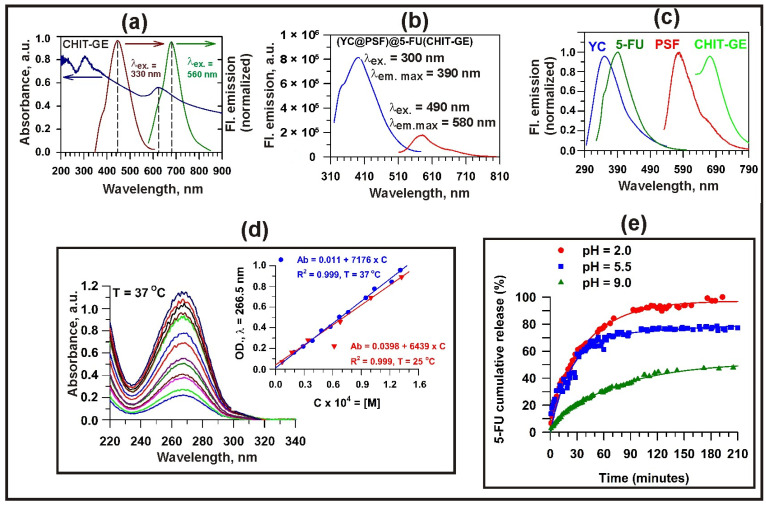
Spectroscopic characterization and 5-FU release profile of (YC@PSF)@5-FU(CHIT–GE) microcapsules. (**a**) UV-Vis spectrum of CHIT–GE in water (blue curve) and its normalized fluorescence emission spectra recorded at excitation wavelengths of 330 nm (brown curve) and 560 nm (green curve). (**b**) Emission spectra of (YC@PSF)@5-FU(CHIT–GE) recorded at excitation wavelengths of 300 nm (blue curve) and 490 nm (red curve). (**c**) Normalized fluorescence emission spectra of native YCs (blue curve) and 5-FU (green curve), along with (YC@PSF)@5-FU(CHIT–GE) microcapsules, showing contributions from encapsulated PSF (red curve) and the CHIT–GE coating (light green curve). (**d**) UV spectra of 5-FU dissolved in water at pH 5.5 and 37 °C. The molar extinction coefficient (ε) of 5-FU was determined to be 6439 M^−1^ cm^−1^ at 25 °C and 7176 M^−1^ cm^−1^ at 37 °C. (**e**) The 5-FU release percentage from (YC@PSF)@5-FU(CHIT–GE) microcapsules was evaluated at 37 °C in distilled water adjusted to pH 2.0 (red circles), 5.5 (blue squares), and 9.0 (green triangles) over specific time intervals.

The fluorescence properties of the CHIT–GE bioconjugate and (YC@PSF)@5-FU(CHIT–GE) microcapsules are shown in Figure 8. As shown in Figure 8a, CHIT–GE exhibits strong fluorescence emission with maxima at 454 nm (λ_ex_ = 330 nm) and 678 nm (λ_ex_ = 560 nm). Figure 8b shows the fluorescence emission spectra of (YC@PSF)@5-FU(CHIT–GE) microcapsules suspended in water over a broad wavelength range (310–800 nm). Upon excitation at λ_ex_ = 300 nm and λ_ex_ = 490 nm, the microcapsules exhibit a YC signal at λ_em_ = 350 nm and an encapsulated 5-FU signal at λ_em_ = 410 nm, which overlaps with the emission of CHIT–GE coating. The PSF emission at 590 nm is accompanied by the weaker CHIT–GE emission at λ_em_ = 678 nm. Figure 8c shows normalized fluorescence emission spectra of native YCs and 5-FU, along with the emission spectra of encapsulated PSF and CHIT–GE coating of (YC@PSF)@5-FU(CHIT–GE) microcapsules. Figure 8d shows the UV spectra of 5-FU in water in the concentration range from 1.0 × 10^−4^ to 1.6 × 10^−4^ M at pH 5.5, used to determine its molar extinction coefficient at 37 °C (ε = 7176 M^−1^ cm^−1^) and 25 °C (ε = 6439 M^−1^ cm^−1^).

The release profiles of 5-FU from (YC@PSF)@5-FU(CHIT–GE) (containing 1.0 mM of drug per gram of microcapsules), shown in Figure 8e, were evaluated over 24 h at 37 °C in water at different pH levels (2.0, 5.5, 9.0). The fastest release occurred within the first three hours, with the initial 5-FU release rate highest during the first 60 min. Approximately 75% of 5-FU was released at pH 2.0, and 68% at pH 5.5 within 60 min, whereas release at pH 9.0 was slower, reaching 50%. The release rate of 5-FU from (YC@PSF)@5-FU(CHIT–GE) microcapsules incubated in an acidic aqueous medium at pH 2.0 is slightly higher than at pH 5.5. However, in a basic aqueous medium at pH 9.0, the drug solubility is lower due to the relatively hydrophobic character of 5-FU, which is sparingly soluble in distilled water at pH 6.5 (approximately 11 g/L). Under basic conditions, 5-FU may precipitate and form dimers and larger aggregates through hydrogen bonding interactions, further reducing its solubility. Moreover, 5-FU is negatively charged (pKa ≈ 8.0); thus, under alkaline conditions, the amine groups of CHIT–GE become deprotonated, leading to a loss of positive charge and the increased compactness of the polymer coating. This reduces electrostatic interactions, while enhanced intra- and intermolecular hydrogen bonding within the polymer may promote chain association. These effects, together with the reduced solubility of 5-FU, may further contribute to drug precipitation and limited release.

#### 3.4.2. Biological Assays of Cytotoxicity of 5-FU Encapsulated in (YC@PSF)@5-FU(CHIT–GE)

**Figure 9:**
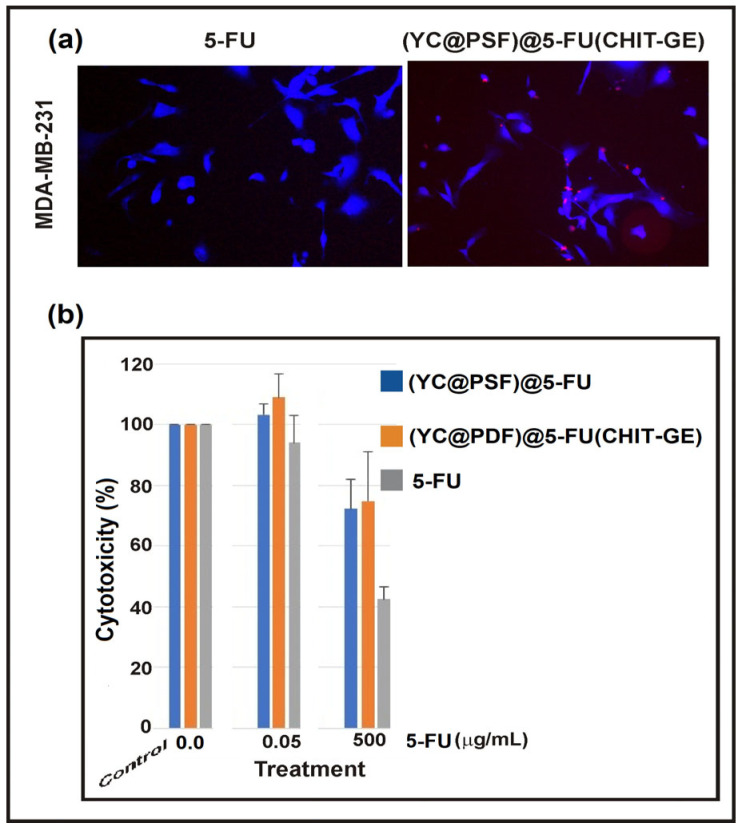
(**a**) The confocal microscopy image confirms uptake of (YC@PSF)@5-FU(CHIT–GE) by MDA-MB-231 cells. (**b**) The graph illustrates the antiproliferative activity of 5-FU against the MDA-MB-231 breast cancer cell line. At a drug concentration of 500 µg/mL, cell viability was 45% for the crude drug and increased to 70% and 75%, respectively, for (YC@PSF)@5-FU and (YC@PSF)@5-FU(CHIT–GE). Reference samples represent cells without 5-FU and 100% viability. The reported data are presented as the mean (±SD) for three independent measurements (n = 3), with statistical significance set at (*p* < 0.0005).

In vitro studies on the triple-negative breast cancer cell line MDA-MB-231 after 72 h demonstrated the anticancer activity of encapsulated 5-FU. Microscopic analysis confirmed the phagocytosis of microcapsules by MDA-MB-231 cells, indicating the successful uptake of the (YC@PSF)@5-FU(CHIT–GE) formulation (Figure 9a).

The antiproliferative activity of encapsulated 5-FU at a concentration of 500 µg/mL (containing 1.0 mM of drug per gram of microcapsules) against cancer cells was lower than that of the native drug, with estimated values of 30%, 25%, and 55% for (YC@PSF)@5-FU, (YC@PSF)@5-FU(CHIT–GE), and 5-FU, respectively (Figure 9b). This reduced effectiveness results from the lower bioavailability of the encapsulated drug within the cell environment. The in vitro experiments carried out for 72 h indicate that the release of 5-FU from the microcapsules may not be complete due to complex interactions between the encapsulated 5-FU and the (YC@PSF)@5-FU(CHIT–GE) polymer network under conditions mimicking the physiological environment (pH 7.4). Moreover, the microenvironment around cells is heterogeneous, with limited fluid dynamics and restricted mass transport, which can slow down drug diffusion from the microcapsules. Additionally, the amine groups of the CHIT–GE polymer matrix in (YC@PSF)@5-FU(CHIT–GE) microcapsules undergo partial deprotonation at pH 7.4, leading to increased compactness and stronger intra- and intermolecular hydrogen bonding. This can hinder capsule swelling and reduce drug diffusion.

## 4. Conclusions

This paper presents a novel and straightforward method for synthesizing microcapsules (YC@PSF)@5-FU(CHIT–GE) with the fluorescent polysaccharide wall on pre-treated yeast cells. In the mechanochemical approach, GE reacts with CHIT to form the CHIT–GE bioconjugate, which exhibits fluorescent properties. Consequently, the outer membrane of (YC@PSF)@5–FU(CHIT–GE) microcapsules is primarily stabilized by electrostatic interactions, hydrogen bonds, and covalent bonds between CHIT–GE and primary amine groups of PSF via GE-mediated cross-linking. These findings provide a rational basis for the design and optimization methods for synthesizing amine-containing microcapsules, enabling the reinforcement of their outer walls via the cross-linking of the envelope with a fluorescent coating. The CLSM analysis confirmed the phagocytosis of (YC@PSF)@5-FU(CHIT–GE) by MDA-MB-231 cells, while in vitro studies revealed the antiproliferative activity of the encapsulated 5-FU.

## Figures and Tables

**Table 1:** Changes in particle size (diameter) distribution measured by DLS, as well as changes in zeta potential for YC, YC@PSF, (YC@PSF)@5-FU, and (YC@PSF)@5-FU(CHIT–GE) microcapsules after 24, 48, and 72 h of incubation in an aqueous environment at 37 °C. Data represent mean ± SD, n =3.

Samples	Particle Size (nm)			Zeta Potential (mV)		
	24 h	48 h	72 h	24 h	48 h	72 h
YC	4891 ± 0.218	4415 ± 0.16	4003 ± 0.37	−5.09 ± 7.82	−4.86 ± 4.30	−4.47 ± 6.89
YC@PSF	3557 ± 0.38	3541 ± 0.18	3488 ± 0.22	−3.40 ± 6.04	−3.08 ± 6.89	−3.76 ± 3.77
(YC@PSF)@5-FU	3891 ± 0.15	3719 ± 0.47	3596 ± 0.49	−10.4 ± 6.25	−4.34 ± 8.29	−4.28 ± 8.01
(YC@PSF)@5-FU(CHIT-GE)	5408 ± 0.12	5384 ± 0.22	5145 ± 0.065	0.582 ± 3.17	−0.793 ± 5.48	−1.08 ± 4.15

**Table 2:** Presents the contact angle (θ) values for films derived from the YC–shell, YC@PSF, and (YC@PSF)@5-FU(CHIT–GE).

Measurements	YC		YC@PSF		(YC@PSF)@5-FU(CHIT–GE)	
	Water	Glycerol	Water	Glycerol	Water	Glycerol
Contact Angle (Average) [degree]	75.5	72.8	90.5	89.5	48.5	43.4
Wetting Energy [mN/m]	18.1	21.1	−0.75	0.64	47.9	52.9
Spreading Coefficient [mN/m]	54.7	51.3	73.6	72.1	24.8	19.8
Work of Adhesion [mN/m]	90.9	94.2	72.0	73.4	120.7	125.7

## Data Availability

The original contributions presented in this study are included in the article. Further inquiries can be directed to the corresponding author.

## References

[1] Soto E.R., Ostroff G.R. (2008). Characterization of multilayered nanoparticles encapsulated in yeast cell wall particles for DNA delivery. Bioconjug. Chem..

[2] Tan Y., Chen L., Li K., Lou B., Liu Y., Liu Z. (2022). Yeast as a carrier for drug delivery and vaccine construction. J. Control. Release.

[3] de Moura I.A., Silva A.J.D., de Macêdo L.S., de Melo K.M.T.B., Leal L.R.S., Espinoza B.C.F., Invenção M.d.C.V., de Pinho S.S., de Freitas A.C. (2025). Advances in the functionalization of vaccine delivery systems: Innovative strategies and translational perspectives. Pharmaceutics.

[4] Wu Y., Li P., Jiang Z., Sun X., He H., Yan P., Xu Y., Liu Y. (2023). Bioinspired yeast-based β-glucan system for oral drug delivery. Carbohydr. Polym..

[5] Tan C., Huang M., McClemens D.J., Sung B., Wang J. (2021). Yeast cell-derived delivery systems for bioactives. Trends Food Sci. Technol..

[6] Zhang X., Xu X., Chen Y., Dou Y., Zhou X., Li L., Li C., An H., Tao H., Hu H. (2017). Bioinspired yeast microcapsules loaded with self-assembled nanotherapies for targeted treatment of cardiovascular disease. Mater. Today.

[7] Liu Y., Wu Q., Wu X., Algharib S.A., Gong F., Hu J., Luo W., Zhou M., Pan Y., Yan Y.Y. (2021). Structure, preparation, modification, and bioactivities of β-glucan and mannan from yeast cell wall: A review. Int. J. Biol. Macromol..

[8] Cabib E., Arroyo J. (2013). How carbohydrates sculpt cells: Chemical control of morphogenesis in the yeast cell wall. Nat. Rev. Microbiol..

[9] Osumi M. (1998). The ultrastructure of yeast: Cell wall structure and formation. Micron.

[10] Li W., Wang H., Xu X.G., Yu Y. (2020). Simultaneous nanoscale imaging of chemical and architectural heterogeneity on yeast cell wall particles. Langmuir.

[11] Klis F.M., Boorsma A., De Groot P.W.J. (2006). Cell wall construction in *Saccharomyces cerevisiae*. Yeast.

[12] Orlean P. (2012). Architecture and biosynthesis of the *Saccharomyces cerevisiae* cell wall. Genetics.

[13] Powell C.D., Quain D.E., Smart K.A. (2003). Citin scar breaks in aged *Saccharomyces cerevisiae*. Microbiology.

[14] Hong F., Yan J., Baran J.T., Allendorf D.J., Hansen R.D., Ostroff G.R., Xing P.X., Cheung N.-K.V., Ross G.D. (2004). Mechanism by Which Orally Administered β-1,3-Glucans Enhance the Tumoricidal Activity of Antitumor Monoclonal Antibodies in Murine Tumor Models. J. Immunol..

[15] Han B., Baruah K., Cox E., Vanrompay D., Bossier P. (2020). Relationship of β-glucans from the perspective of immunomodulation: A mini-review. Front. Immunol..

[16] Liu D.-q., Lu S., Zhang L.-x., Ji M., Liu S.-y., Wang S.-w., Liu R.-t. (2018). An indoleamine 2,3-dioxygenase siRNA nanoparticle-coated and Trp2-displayed recombinant yeast vaccine inhibits melanoma tumor growth in mice. J. Control. Release.

[17] Mi Y., Hagan T., Vincent B.G., Wang A.Z. (2019). Emerging nano-/microapproaches for cancer immunotherapy. Adv. Sci..

[18] Zhou X., Zhang X., Han S., Dou Y., Liu M., Zhang L., Guo J., Shi Q., Gong G., Wang R. (2017). Yeast microcapsule-mediated targeted delivery of diverse nanoparticles for imaging and therapy via the oral route. Nano Lett..

[19] Li X., Zhao Z., Yang Y., Liu Z., Wang J., Xu Y., Zhang Y. (2020). Novel β-1,3-D-glucan porous microcapsule enveloped folate-functionalized liposomes as a Trojan horse for facilitated oral tumor-targeted co-delivery of chemotherapeutic drugs and quantum dots. J. Mater. Chem. B.

[20] Ren T., Gou J., Sun W., Tao X., Tan X., Wang P., Zhang Y., He H., Yin T., Tang X. (2018). Entrapping of nanoparticles in yeast cell wall microparticles for macrophage-targeted oral delivery of cabazitaxel. Mol. Pharm..

[21] Upadhyay T.K., Fatima N., Sharma D., Saravanakumar V., Sharma R. (2017). Preparation and characterization of beta-glucan particles containing a payload of nanoembedded rifabutin for enhanced targeted delivery to macrophages. EXCLI J..

[22] Soto E., Caras A.C., Kut L.C., Castle M.K., Ostroff G.R. (2012). Glucan particles for macrophage targeted delivery of nanoparticles. J Drug Deliv..

[23] Tan C., Wang J., Sun B. (2021). Polysaccharide dual coating of yeast capsules for stabilization of anthocyanins. Food Chem..

[24] Lee K., Kwon Y., Hwang J., Choi Y., Kim K., Koo H.-J., Seo Y., Jeon H., Choi J. (2019). Synthesis and Functionalization of β-Glucan Particles for the Effective Delivery of Doxorubicin Molecules. ACS Omega.

[25] Sadeghi A., Ebrahimi M., Shahryari S., Assadpour E., Jafari S.M. (2024). Potential applications of encapsulated yeasts especially within alginate and chitosan as smart bioreactors and intelligent micro-machines. Carbohydr. Polym. Technol. Appl..

[26] Shi G., Liu Y., He Z., Zhou J. (2017). Chemical treatment and chitosan coating of yeast cells to improve the encapsulation and controlled release of bovine serum albumin. Artif. Cells Nanomed. Biotechnol..

[27] Anjum S., Naseer F., Ahmad T., Jahan F., Qadir H., Gul R., Kousar K., Sarwar A., Shabbir A. (2024). Enhancing therapeutic efficacy: Sustained delivery of 5-fluorouracil (5-FU) via thiolated chitosan nanoparticles targeting CD44 in triple-negative breast cancer. Sci. Rep..

[28] Muzzarelli R.A.A., Mehtedi M.E., Bottegoni C., Aquili A., Gigante A. (2015). Crosslinked chitosan gel and scaffolds for tissue engineering and regeneration of cartilage and bone. Mar. Drugs.

[29] Pujana M.A., Pérez-Álvarez L., Iturbe L.C.C., Katime I. (2013). Biodegradable chitosan nanogels crosslinked with genipin. Carbohydr. Polym..

[30] Ranganath S.H., Tan A.L., He F., Wang C.-H., Krantz W.B. (2011). Control and enhancement of perm-selectivity of membrane-based microcapsules for favorable biomolecules transport and immunoisolation. AIChE J..

[31] Trzeciak K., Dudek M.K., Potrzebowski M.J. (2024). Mechanochemical transformations of pharmaceutical cocrystals: Polymorphs and conformer exchange. Chem. Eur. J..

[32] Jin J., Song M., Hourston D.J. (2004). Novel chitosan-based films cross-linked by genipin with improved physical properties. Biomacromolecules.

[33] González A., Strumia M.C., Alvarez Igarzabal C.I. (2011). Cross-linked soy protein as material for biodegradable films: Synthesis, characterization, and biodegradation. J. Food Eng..

[34] Mi F.-L., Sung H.-W., Shyu S.-S. (2000). Synthesis and characterization of a novel chitosan-based network prepared using naturally occurring crosslinker. J. Polym. Sci. Part A Polym. Chem..

[35] Mi L. (2005). Synthesis and characterization of a novel chitosan-gelatin bioconjugate with fluorescence emission. Biomacromolecules.

[36] Li Q., Wang X., Lou X., Yuan H., Tu H., Li B., Zhang Y. (2015). Genipin-crosslinked electrospun chitosan nanofibers: Determination of crosslinking conditions and evaluation of cytocompatibility. Carbohydr. Polym..

[37] Jardim K.V., Joanitti G.A., Azevedo R.B., Parize A.L. (2015). Physico-chemical characterization and cytotoxicity evaluation of curcumin loaded in chitosan/chondroitin sulfate nanoparticles. Mater. Sci. Eng. C.

[38] Dimida S., Barca A., Cancelli N., De Benedictis V., Raucci M.G., Demitri C. (2017). Effect of genipin concentration on cross-linked chitosan scaffold for bone tissue engineering: Structural characterization and evidence of biocompatibility features. Int. J. Polym. Sci..

[39] Mi F.-L., Shyu S.-S., Peng C.-K. (2005). Characterization of ring-openung polymerization of genipin and pH-dependent cross-linking reactions between chitosan and genipin. J. Polym. Sci. Part A Polym. Chem..

[40] He L., Du J., Wang J., Tan C. (2026). Innovative yeast-based delivery systems for enhancing bioaccessibility and bioavailability of bioactive compounds. Trends Food Sci. Technol..

[41] Glasman J.D., Alaimo A., López C.S., Farías M.E., Currá R.B., Lamas D.G., Pérez O.E. (2025). Perspectives on the pH-influenced design of chitosan-genipin nanogels for cell-targeted delivery. Pharmaceutics.

[42] Miras J., Liu C., Blomberg E., Thormann E., Vílchez S., Esquena J. (2021). pH-Responsive chitosan nanofilms crosslinked with genipin. Colloid. Surf. A Physicochem. Eng. Asp..

[43] Mei M., Bai B., Zheng D., Hu N., Wang H. (2021). Novel fabrication of a yeast biochar-based photothermal-responsive platform for controlled imidacloprid release. RSC Adv..

[44] Neto C.G.T., Giacometti J.A., Job A.F., Ferreira F.C., Fonseca J.L.C., Pereira M.R. (2005). Thermal analysis of chitosan polymers. Carbohydr. Polym..

